# Neurotoxicity of Sri Lankan Krait (*Bungarus ceylonicus*) and Common Krait (*Bungarus caeruleus*) Venoms and Their Neutralisation by Commercial Antivenoms In Vitro

**DOI:** 10.3390/toxins17090439

**Published:** 2025-09-02

**Authors:** Jithmi Galappaththige, Geoffrey K. Isbister, Kalana Maduwage, Wayne C. Hodgson, Anjana Silva

**Affiliations:** 1 Department of Parasitology, Faculty of Medicine and Allied Sciences, Rajarata University of Sri Lanka, Saliyapura 50008, Sri Lanka; jithmidinu@gmail.com; 2 Department of Biomedical Sciences, ICBT Campus, Colombo 00400, Sri Lanka; 3 Clinical Toxicology Research Group, University of Newcastle, Waratah, NSW 2298, Australia; geoff.isbister@gmail.com; 4 South Asian Clinical Toxicology Research Collaboration, University of Peradeniya, Peradeniya 20400, Sri Lanka; 5 School of Medicine and Dentistry, Griffith University, Gold Coast, QLD 4222, Australia; kalanapm@gmail.com; 6 Monash Venom Group, Department of Pharmacology, Faculty of Medicine, Nursing and Health Sciences, Monash University, Melbourne, VIC 3800, Australia; wayne.hodgson@monash.edu

**Keywords:** *Bungarus*, Sri Lankan krait, neurotoxicity, post-synaptic, pre-synaptic, antivenoms

## Abstract

The common krait (*Bungarus caeruleus*) and the endemic Sri Lankan krait (*B. ceylonicus*) are two species of krait responsible for envenomings in Sri Lanka that result in progressive neuromuscular paralysis. We characterised the in vitro neurotoxicity of *B. ceylonicus* and *B. caeruleus* venoms and studied their neutralisation by two commercially available Indian polyvalent antivenoms (i.e., VINS and BHARAT), Thai banded krait antivenom and Australian polyvalent antivenom using the chick biventer cervicis nerve-muscle preparation. Both venoms displayed concentration-dependent neurotoxicity, showing equipotent pre-synaptic neurotoxicity at 0.03 μg/mL. At a higher concentration (1 μg/mL), both venoms showed post-synaptic neurotoxicity, with *B. ceylonicus* venom being more potent. VINS was unable to neutralise the neurotoxicity of *B. ceylonicus* venom, but neutralised both pre- and post-synaptic neurotoxicity of *B. caeruleus* venom. BHARAT neutralised in vitro pre- and post-synaptic activity of both *B. ceylonicus* and *B. caeruleus* venoms. Banded krait antivenom and Australian polyvalent antivenoms were unable to fully neutralise the neurotoxicity of either venom at tested concentrations. In conclusion, *B. ceylonicus* venom shows pre- and post-synaptic neurotoxicity similar to *B. caeruleus* venom. BHARAT effectively neutralises both pre- and post-synaptic neurotoxicity of *B. ceylonicus* venom. Both Indian polyvalent antivenoms effectively neutralise neurotoxicity induced by *B. caeruleus* venom.

## 1. Introduction

Envenoming by kraits (Genus: *Bungarus*) is a significant healthcare problem in South and Southeast Asia [1,2]. Krait envenoming results in progressive neuromuscular paralysis in humans due to the blockade of neurotransmission by toxins acting at the neuromuscular junction (NMJ) [3]. The most clinically important neurotoxins in krait venoms in relation to human envenoming are the beta-bungarotoxins. These are phospholipase A_2_ (PLA_2_) toxins that act pre-synaptically at the NMJ [4]. Beta-bungarotoxins initially hydrolyse the phospholipids in the neurilemma of the motor nerve terminal, followed by the depletion of synaptic vesicles and degeneration of the mitochondria, causing a denervation leading to a treatment-resistant paralysis in humans [4,5,6]. In addition, krait venoms possess α-neurotoxins that act post-synaptically at the NMJ. Alpha-neurotoxins belong to the three-finger toxin family and are of two main types, i.e., short- and long-chain α-neurotoxins. These neurotoxins competitively bind with the agonist binding sites of nicotinic acetylcholine receptors (nAChR) located in the post-synaptic membrane in the neuromuscular junction, thereby blocking neuromuscular transmission [7]. However, the clinical relevance of α-neurotoxins, particularly the short-chain neurotoxins, to paralysis in humans has been challenged [8].

Sri Lanka is a global snakebite hotspot with both viperids and elapids being responsible for significant mortality and morbidity. Two species of kraits are found in Sri Lanka. The more widespread of the two, the common Krait/Indian krait (*Bungarus caeruleus*), is distributed throughout the dry and intermediate zones of Sri Lanka, in addition to many parts of South Asia. Whereas the Sri Lankan krait (*Bungarus ceylonicus*) is an endemic species restricted to the wet zone and intermediate zones of Sri Lanka [9]. Although the composition of Sri Lankan *B. caeruleus* venom has been previously studied, the composition and pharmacology of Sri Lankan *B. ceylonicus* venom remain unexplored to date [10,11]. Envenoming by *B. caeruleus* is common in the dry zone of Sri Lanka, particularly during the monsoon, while envenoming by *B. ceylonicus* is limited to scattered reports from the wet zone of Sri Lanka [12,13]. Similar to other krait species, envenoming by both *B. caeruleus* and *B. ceylonicus* results in progressive neuromuscular paralysis, including life-threatening respiratory paralysis [14,15].

The only available snake antivenom in Sri Lanka is Indian polyvalent antivenom, which is developed against the venoms of Russell’s viper (*Daboia russelii*), cobra (*Naja naja*), saw-scaled viper (*Echis carinatus*), as well as the common krait (*B. caeruleus*). In Sri Lanka, patients envenomed by either *B. caeruleus* or *B. ceylonicus* are treated with Indian polyvalent antivenom [13,16]. However, the ability of Indian polyvalent antivenom to cross-neutralise the most important clinical effect of *B. ceylonicus* venom, i.e., neurotoxicity, has not been previously investigated.

This study aims to pharmacologically characterise the in vitro neurotoxicity of Sri Lankan *B. ceylonicus* and *B. caeruleus* venoms, and to test the efficacy of Indian polyvalent antivenom, Thai banded krait antivenom and Australian polyvalent antivenom to cross-neutralise their neurotoxicity.

## 2. Results

### 2.1. Concentration-Dependent Neurotoxicity of B. ceylonicus and B. caeruleus Venoms

**Figure 1:**
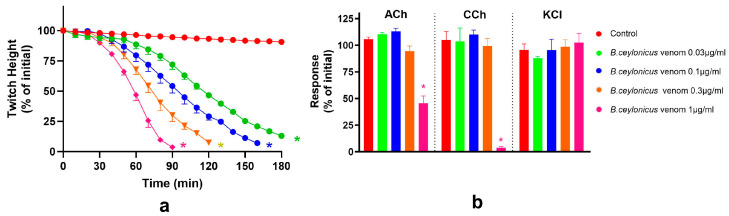
(**a**) Concentration-dependent inhibition of indirect twitches of the chick biventer cervicis nerve-muscle preparation by *B. ceylonicus* venom; (**b**) Effect of *B. ceylonicus* venom on contractile responses to ACh, CCh or KCl (* significantly different from control, *p* < 0.05, Unpaired *t*-test n = 3–6). Error bars indicate the standard error of the mean.

*B. ceylonicus* venom caused concentration-dependent inhibition of the indirect twitches of the chick biventer cervicis nerve-muscle preparation, with the time taken for 50% inhibition of the twitch height (t50) decreasing with the increase of venom concentration (Table 1). *B. ceylonicus* venom (0.03, 0.1 and 0.3 μg/mL) did not significantly inhibit contractile responses to ACh, CCh, or KCl, indicating a pre-synaptic mode of action (Figure 1). *B. ceylonicus* venom (1 μg/mL) significantly inhibited the response to ACh and abolished the response to CCh, without reducing the response to KCl, indicating a post-synaptic mode of action (Figure 1).

The minimum concentration of *B. ceylonicus* venom that displayed a selective pre-synaptic mode of action (i.e., 0.03 μg/mL) was used for subsequent experiments to examine the efficacy of antivenoms on the pre-synaptic effects of the venom. The minimum concentration of *B. ceylonicus* venom that displayed a post-synaptic mode of action (i.e., 1 μg/mL) was selected for subsequent experiments on antivenom efficacy on post-synaptic effects of the venom.

**Figure 2:**
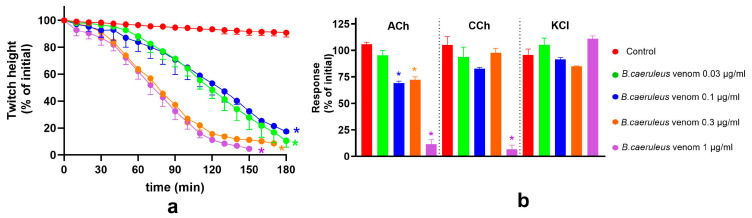
(**a**) Concentration-dependent inhibition of indirect twitches in the chick biventer cervicis nerve-muscle preparation by *B. caeruleus* venom; (**b**) Effect of *B. caeruleus* venom on contractile responses to ACh, CCh or KCl (* significantly different from control, *p* < 0.05, unpaired *t*-test, n = 3–6). Error bars indicate the standard error of the mean.

*B. caeruleus* venom caused concentration-dependent inhibition of indirect twitches of the chick biventer cervicis nerve-muscle preparation, with the time taken for 50% inhibition of the twitch height (i.e., t_50_) decreasing with the increase of venom concentration (Table 1). *B. caeruleus* venom (0.03, 0.1, and 0.3 μg/mL) concentrations did not inhibit the contractile response of the muscle towards the exogenous agonists CCh and KCl, indicating a pre-synaptic mode of action (Figure 2). Although there was a slight reduction in the response to ACh at 0.1 and 0.3 μg/mL, in contrast, *B. caeruleus* venom (1 μg/mL) markedly inhibited the contractile response of the muscle to ACh and CCh, without significantly inhibiting the response to KCl, indicating a post-synaptic mode of action.

The minimum concentration of *B. caeruleus* venom that displayed a selective pre-synaptic mode of action (0.03 μg/mL) was used for subsequent experiments examining the efficacy of antivenoms on the pre-synaptic effects of the venom. The minimum concentration of *B. caeruleus* venom that displayed a selective post-synaptic mode of action (1 μg/mL) was used to examine the efficacy of antivenoms on the post-synaptic effects of the venom.

In comparison, both venoms displayed similar potency in pre-synaptic activity at 0.03 μg/mL, with both failing to achieve 90% inhibition of twitch height (t_90_) while the t_50_ of *B. ceylonicus* and *B. caeruleus* venoms were similar (*p* > 0.05, unpaired *t*-test; Table 1). However, *B. ceylonicus* (1 μg/mL) was more potent with regard to post-synaptic activity, with t_90_ being 79.7 (SD, 4.0) min and t_90_ of *B. caeruleus* venom being 129.3 (SD, 2.1) min (*p* < 0.001, unpaired *t*-test, Table 1).

### 2.2. Effect of Indian Polyvalent Antivenoms on the Pre-Synaptic and Post-Synaptic Activity of B. ceylonicus Venom

#### 2.2.1. Pre-Synaptic Activity of *B. ceylonicus* Venom

The ability of VINS and BHARAT antivenoms (×1 and ×10 concentrations) to neutralise the pre-synaptic activity of *B. ceylonicus* venom (0.03 μg/mL) in the chick-biventer cervicis nerve-muscle preparation was examined.

**Figure 3:**
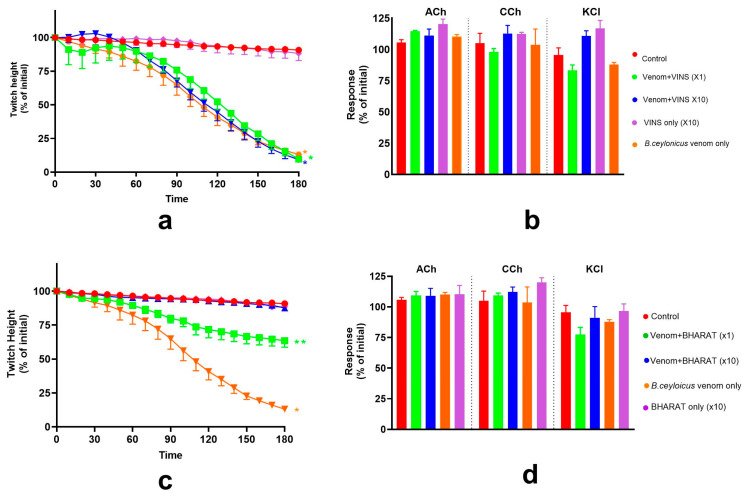
The effect of Indian polyvalent antivenoms (×1 and ×10 concentrations) on the *B. ceylonicus* venom (0.03 μg/mL) pre-synaptic neurotoxicity: (**a**,**c**) The effect of VINS or BHARAT antivenoms (AV) on the venom-mediated inhibition of the indirect twitches of the chick-biventer cervicis nerve-muscle preparation; (**b**,**d**) The effect of the VINS or BHARAT AV, in the presence of venom, on contractile responses to ACh, CCh or KCl. (* significantly different from control; ** significantly different from control and venom alone; *p* < 0.05, one-way ANOVA followed by Tukey’s multiple comparison test, n = 3–6). Error bars indicate the standard error of the mean.

Neither concentration of VINS antivenom neutralised the pre-synaptic neurotoxicity of *B. ceylonicus* venom (0.03 μg/mL; Figure 3). The pre-synaptic neurotoxicity of *B. ceylonicus* venom (0.03 μg/mL) was partially neutralised by the ×1 concentration of BHARAT antivenom and fully neutralised by the ×10 concentration of antivenom (Figure 3), as evidenced by the lack of twitch inhibition caused by the venom in the presence of ×10 antivenom.

#### 2.2.2. Post-Synaptic Activity of *B. ceylonicus* Venom

**Figure 4:**
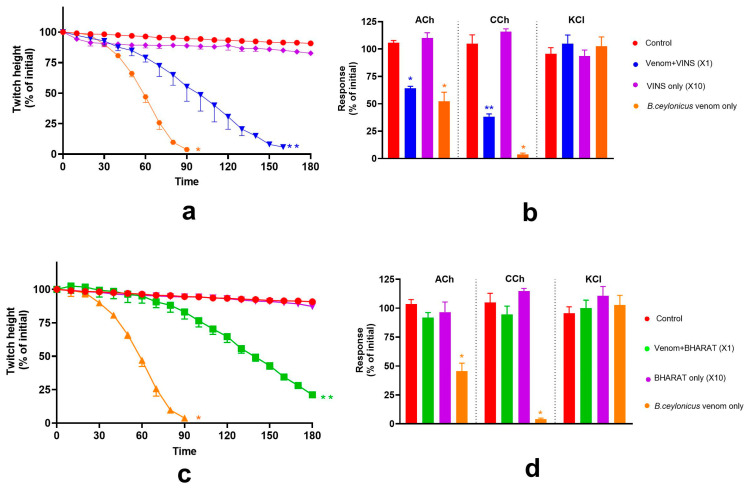
Effect of Indian polyvalent antivenoms (either ×1 or ×10) on *B. ceylonicus* venom (1 μg/mL) mediated post-synaptic neurotoxicity: (**a**,**c**) The effect of VINS and BHARAT antivenoms (AV) on the venom-mediated inhibition of the indirect twitches of the chick-biventer cervicis nerve-muscle preparation; (**b**,**d**) The effect of VINS and BHARAT AV on contractile responses to ACh, CCh or KCl, in the presence of venom. (* significantly different from control; ** significantly different from control and venom alone; *p* < 0.05, one-way ANOVA followed by Tukey’s multiple comparison test, n = 3–6). Error bars indicate the standard error of the mean.

The effect of VINS and BHARAT antivenoms on the post-synaptic activity of the *B. ceylonicus* venom (1 μg/mL) on the chick-biventer cervicis nerve-muscle preparation was examined. VINS antivenom (×1) partially neutralised the post-synaptic neurotoxicity of *B. ceylonicus* venom (1 μg/mL; Figure 4), whereas BHARAT antivenom (×1) fully neutralised the post-synaptic neurotoxicity of *B. ceylonicus* venom (1 μg/mL; Figure 4).

### 2.3. Effect of Thai Banded Krait Antivenom and Australian Polyvalent Antivenom on the Pre-Synaptic and Post-Synaptic Activity of B. ceylonicus Venom

**Figure 5:**
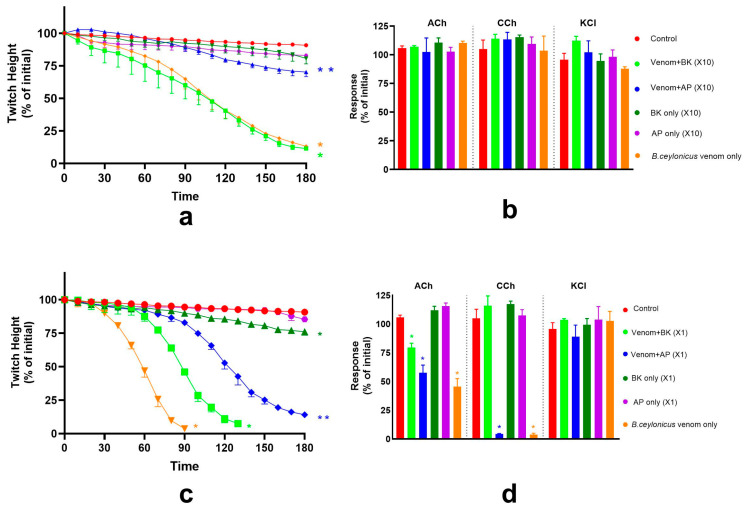
The effect of Thai banded krait (BK) or Australian polyvalent (AP) antivenoms (AV; each at ×10 recommended concentration) on pre- and post-synaptic neurotoxicity of the *B. ceylonicus* venom: (**a**) The effect of the AV (×10) on *B. ceylonicus* venom (0.03 μg/mL) mediated inhibition of the indirect twitches of the chick-biventer cervicis nerve-muscle preparation (* significantly different from control at the corresponding time, ** significantly different from control and venom. *p* < 0.05, one-way ANOVA followed by Tukey’s multiple comparison tests); (**b**) The effects of AV (×10) on contractile responses to ACh, CCh or KCl, in the presence of *B. ceylonicus* venom (0.03 μg/mL) (* significantly different from control, *p* < 0.05, one-way ANOVA followed by Tukey’s multiple comparison test); (**c**) The effect of antivenoms (each at ×1) on *B. ceylonicus* venom (1 μg/mL) mediated inhibition of the indirect twitches of the chick biventer cervicis nerve-muscle muscle preparation (* significantly different from control at the corresponding time, ** significantly different from control and venom. *p* < 0.05, one-way ANOVA followed by Tukey’s multiple comparison test); (**d**) The effect of the antivenoms (each at ×1) on the contractile response of the muscle induced by exogenous agonists, ACh, CCh or KCl, in the presence of 1 μg/mL *B. ceylonicus* venom. (* significantly different from control, *p* < 0.05, one-way ANOVA followed by Tukey’s multiple comparison test). Error bars indicate the standard error of the mean; n = 3–6.

The efficacy of Thai banded krait or Australian polyvalent antivenom (each at ×10) against the pre-synaptic activity of *B. ceylonicus* venom (0.03 μg/mL) on the chick biventer cervicis nerve-muscle preparation was examined. Thai banded krait antivenom did not neutralise, whereas Australian polyvalent antivenom partially neutralised, *B. ceylonicus* venom (0.03 μg/mL) mediated pre-synaptic neurotoxicity (Figure 5a,b).

The efficacy of Thai banded krait or Australian polyvalent antivenom (each at ×1) against the post-synaptic activity of *B. ceylonicus* venom (1 μg/mL) on the chick-biventer cervicis nerve-muscle preparation was examined. Neither Thai banded krait antivenom nor Australian polyvalent antivenom fully neutralised the post-synaptic neurotoxicity of the venom. However, both antivenoms significantly delayed venom-mediated inhibition of the indirect twitches of the preparation (Figure 5c,d).

### 2.4. Effect of Indian Polyvalent Antivenoms on the Pre-Synaptic and Post-Synaptic Activity of B. caeruleus Venom

**Figure 6:**
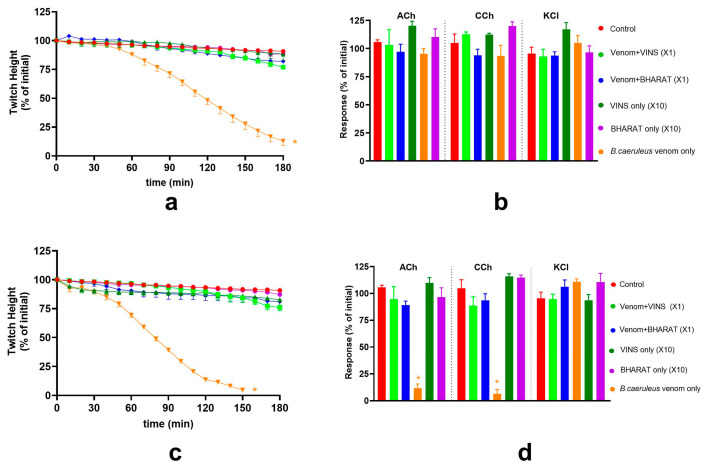
Effect of VINS and BHARAT antivenoms (AV; each at ×1 recommended concentration) on the *B. caeruleus* venom-mediated pre- and post-synaptic neurotoxicity. (**a**) The effect of the antivenoms on *B. caeruleus* venom (0.03 μg/mL) mediated inhibition of indirect twitches of the chick biventer cervicis nerve-muscle preparation. (**b**) The effects of AV on contractile responses of the muscle to ACh, CCh or KCl, in the presence of venom. (**c**) The effect of AV on *B. caeruleus* venom (1 μg/mL) mediated inhibition of the indirect twitches of the chick-biventer cervicis nerve-muscle preparation; (**d**) The effects of the antivenoms on contractile responses of the muscle to ACh, CCh or KCl, in the presence of 1 μg/mL venom. (* significantly different from control; *p* < 0.05, one-way ANOVA followed by Tukey’s multiple comparison test, n = 3–6; Error bars indicate the standard error of the mean).

The efficacy of VINS and BHARAT antivenoms (each at ×1) was examined against the pre-synaptic and post-synaptic activity of *B. caeruleus* venom in the chick-biventer cervicis nerve-muscle preparation. Each antivenom fully neutralised the pre-synaptic activity of the *B. caeruleus* venom (0.03 μg/mL; Figure 6a,b) and the pre- and post-synaptic activity of *B. caeruleus* venom (1 μg/mL; Figure 6c,d).

### 2.5. Effect of Thai Banded Krait and Australian Polyvalent Antivenoms on the Pre-Synaptic and Post-Synaptic Activity of B. caeruleus Venom

**Figure 7:**
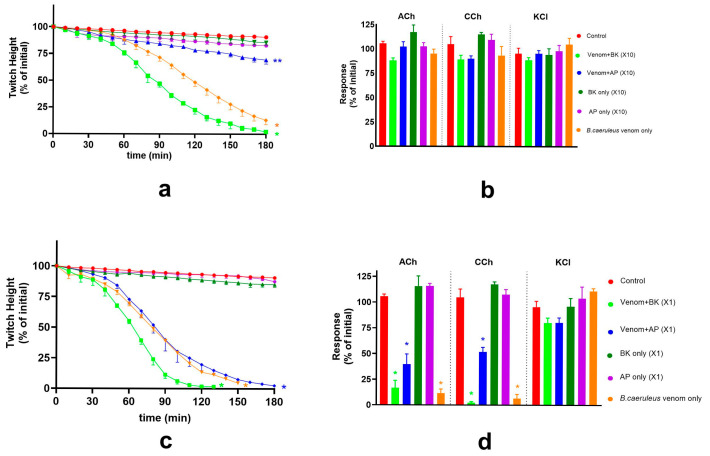
The effect of Thai banded krait (BK) and Australian polyvalent (AP) antivenoms (AV) on pre- and post-synaptic neurotoxicity of *B. caeruleus* venom: (**a**) The effect of the AV (each at ×10 recommended concentration) on *B. caeruleus* venom (0.03 μg/mL) mediated inhibition of indirect twitches of the chick-biventer cervicis nerve-muscle preparation (* significantly different from control at the corresponding time, ** significantly different from control and the venom alone, *p* < 0.05, one-way ANOVA followed by Tukey’s multiple comparison test); (**b**) AV (×10 concentration) on contractile responses to ACh, CCh or KCl, in the presence of *B. caeruleus* venom (0.03 μg/mL) (* significantly different from control, *p* < 0.05, one-way ANOVA followed by Tukey’s multiple comparison test); (**c**) The effect of AV (×1 concentration) on the *B. caeruleus* venom (1 μg/mL) mediated inhibition of indirect twitches of the chick-biventer cervicis nerve-muscle preparation (* significantly different from control at the corresponding time, *p* < 0.05, one-way ANOVA followed by Tukey’s multiple comparison test); (**d**) The effect of AV (×1 concentration) on contractile responses to ACh, CCh or KCl, in the presence of *B. caeruleus* venom (1 μg/mL). (* significantly different from control, *p* < 0.05, one-way ANOVA followed by Tukey’s multiple comparison test). Error bars indicate the standard error of the mean; n = 3–6.

The efficacy of Thai banded krait antivenom and Australian polyvalent antivenom (each at ×10 recommended concentration) was examined against the pre-synaptic activity of *B. caeruleus* venom in the chick biventer cervicis nerve-muscle preparation. Thai banded krait antivenom did not neutralise *B. caeruleus* venom (0.03 μg/mL) mediated pre-synaptic neurotoxicity. Whereas, Australian polyvalent antivenom partially neutralised *B. caeruleus* venom (0.03 μg/mL) mediated pre-synaptic neurotoxicity, as indicated by the marked reduction in inhibition caused by the venom in the presence of antivenom compared to venom alone (Figure 7a,b).

Thai banded krait and Australian polyvalent antivenoms (each at ×1 recommended concentration) to test their effect on the post-synaptic activity of the 1 μg/mL *B. caeruleus* venom on the chick-biventer nerve-muscle preparation. Neither antivenom neutralised the post-synaptic neurotoxicity of the venom (Figure 7c,d).

## 3. Discussion

*B. ceylonicus* and *B. caeruleus* venoms exhibited concentration-dependent in vitro neurotoxicity in the chick biventer cervicis nerve-muscle preparation. Both venoms displayed primarily pre-synaptic neurotoxicity at lower concentrations and post-synaptic neurotoxicity at higher concentrations. The venoms showed similar potency for pre-synaptic activity. However, *B. ceylonicus* displayed more potent post-synaptic activity. The non-specific BHARAT polyvalent antivenom cross-neutralised the clinically important pre-synaptic neurotoxicity, as well as the post-synaptic neurotoxicity of *B. ceylonicus* venom. In addition, the pre- and post-synaptic activities of *B. caeruleus* venom were neutralised by either VINS or BHARAT polyvalent antivenoms at the recommended concentrations. In contrast, Thai banded krait antivenom failed to neutralise the pre- and post-synaptic neurotoxicity of both venoms, while Australian polyvalent antivenom only neutralised the pre-synaptic neurotoxicity of both venoms.

Neuromuscular paralysis, ranging from mild effects such as ptosis and ophthalmoplegia to life-threatening paralysis that includes bulbar and respiratory paralysis, has been reported following *B. ceylonicus* envenoming [12,13,14,16,17]. Due to the absence of a specific homologous antivenom, Indian polyvalent antivenom has been used to treat *B. ceylonicus* envenoming in Sri Lanka despite a lack of experimental or clinical evidence regarding the efficacy of this approach. In this study, we have shown that the BHARAT Indian polyvalent antivenom effectively cross-neutralised the neurotoxicity of *B. ceylonicus* venom, indicating potential for use in *B. ceylonicus* envenoming in Sri Lanka.

In contrast, VINS antivenom was unable to cross-neutralise the neurotoxicity of *B. ceylonicus* venom. However, both BHARAT and VINS antivenoms effectively neutralised Sri Lankan *B. caeruleus* venom-mediated pre- and post-synaptic neurotoxicity at their recommended concentrations, indicating the presence of sufficient antibodies in the two homologous antivenoms to neutralise both types of neurotoxicity. VINS and BHARAT antivenoms have shown remarkable batch-to-batch variation in their efficacy against Sri Lankan snake venoms previously [18]. In the present study, only one batch from each of VINS and BHARAT antivenoms was used; hence, the potential effect of the inter-batch variation of these antivenoms on the efficacy in neutralising neurotoxic effects of *B. ceylonicus* venom cannot be excluded. Thai banded krait antivenom did not cross-neutralise the neurotoxicity of either krait venom, while Australian polyvalent antivenom only partially neutralised the pre-synaptic neurotoxicity of both venoms. A previous proof-of-concept in vitro study using the chick-biventer model showed widespread cross-neutralisation of pre-synaptic and post-synaptic neurotoxins of different Asian and Australian snake venoms by heterologous antivenoms from Asia and Australia [19]. In this previous study, 200 μL of antivenom was used in 5 mL organ baths, while the maximum volume of antivenom used in the present study was 155 μL in 25 mL organ baths (×10 antivenom concentration for 1 μg/mL venom concentrations). Therefore, the maximum concentrations of all antivenoms tested in the present study were significantly lower. Therefore, the failure of heterologous antivenoms to cross-neutralise the two tested krait venoms could be due to the inadequate concentrations of the antivenoms and/or the absence of antibodies to neutralise all pre- and post-synaptic neurotoxins of the two venoms.

This study only tested the ability of the antivenoms to prevent the neurotoxicity caused by the two krait venoms (i.e., to check the availability of the neutralising antibodies against pre- and post-synaptic neurotoxins), by adding the antivenom before the venom into the organ baths. While this is the standard method of testing the in vitro efficacy of antivenoms, it does not mirror the actual snakebite [3,20]. In Sri Lanka, 17 patients with common krait envenoming were admitted to the hospital a median of 3.3 h after the bite and received Indian polyvalent antivenom a median of 3.5 h after the bite [15]. This delay in antivenom enables the neurotoxins to be absorbed into the circulation and reach their target sites, the neuromuscular junctions. In krait envenoming, the clinically most important neurotoxins are the pre-synaptic neurotoxins due to their ability to induce treatment-resistant progressive neuromuscular paralysis [4]. However, it is uncertain whether the antibody fragments, which are much larger molecules than the toxins, can effectively reach neuromuscular junctions before the irreversible activity of the pre-synaptic neurotoxins is initiated [3,20]. A previous study showed that Australian polyvalent antivenom neutralised the activity of the pre-synaptic neurotoxin taipoxin from coastal taipan (*Oxyuranus scutellatus*) venom if the antivenom was added to the chick-biventer nerve-muscle preparation within 15 min of venom addition [21]. This means that even if the antivenoms had antibodies to neutralise the pre-synaptic neurotoxins, their role in the prevention of the life-threatening paralysis could be limited to a very narrow time window. The potential of small molecule therapeutics, such as PLA2 inhibitor varespladib, to be used alone or as adjunct therapy to antivenom in widening this time window of krait venom-induced neuromuscular paralysis has been suggested in experimental studies [22,23].

The lower venom concentrations of both venoms displayed pre-synaptic-predominant neurotoxicity, while the highest venom concentration (1 μg/mL) showed post-synaptic-predominant neurotoxicity in the chick biventer cervicis nerve-muscle preparation. This may indicate that at lower concentrations of the venom, the post-synaptic activity is not potent enough to mask the pre-synaptic activity, while at higher concentrations of venom, the more rapidly acting post-synaptic toxins are able to mask the pre-synaptic neurotoxicity. In such situations, if an antivenom can neutralise only the post-synaptic neurotoxicity, non-neutralised or partially neutralised pre-synaptic neurotoxicity could still be observable [21]. This explains the observation that the BHARAT antivenom, at ×1 concentration, neutralises the post-synaptic neurotoxicity of 1 μg/mL *B. ceylonicus* venom but fails to neutralise the pre-synaptic neurotoxicity of the venom at that concentration.

There are several limitations of this study. Only one batch from each antivenom was used. Hence, the observations may not be readily generalisable for all batches of a particular antivenom. Further, there is a limitation to test higher concentrations of antivenom in organ baths without significantly altering the physiological conditions of the organ baths. Although the antivenom-only controls were used to ensure that the antivenom does not affect the muscle activity of the tested antivenom concentrations, we were not able to test very high antivenom concentrations without significantly altering the bath conditions.

## 4. Conclusions

Both *B. ceylonicus* and *B. caeruleus* venoms show pre-synaptic activity at lower venom concentrations and exhibit post-synaptic activity at higher venom concentrations in vitro. *B. ceylonicus* and *B. caeruleus* venoms have equipotent pre-synaptic neurotoxicity. Pre- and post-synaptic neurotoxicity of *B. ceylonicus* was fully neutralised by the BHARAT Indian polyvalent antivenom, indicating its potential to be used in *B. ceylonicus* envenomed patients. Pre- and post-synaptic neurotoxicity of *B. caeruleus* was fully neutralised by both BHARAT and VINS Indian polyvalent antivenoms. Thai banded krait and Australian polyvalent antivenoms were not able to fully cross-neutralise the two krait venoms at tested concentrations.

## 5. Materials and Methods

### 5.1. Venoms and Antivenoms

Freeze-dried, pooled *B. ceylonicus* and *B. caeruleus* venoms, sourced from multiple wild snakes from each species, dissolved in 50% glycerol, were stored in −80 °C until required.

Two brands of Indian polyvalent antivenom, VINS (Vins Bioproducts limited, Hyderabad, India, Batch No: 01AS21073) and BHARAT (Bharat Serums and Vaccines, Ambernath, India, Batch No: A05322003), Banded Krait antivenom (Queen Savovabha Memorial Institute, Thai Red Cross Society, Bangkok, Thailand; Batch no: BK0112;) and Australian polyvalent antivenom (Seqirus Pty Ltd., Parkville, Australia; Batch No: 055517801) were used. Indian polyvalent and Thai banded krait antivenom vials were reconstituted in 10 mL of distilled water, as per the manufacturer’s instructions. According to the manufacturers, 1 mL of reconstituted Indian polyvalent antivenom neutralises 0.45 mg of common krait (*B. caeruleus*) venom, whereas 1 mL of reconstituted Thai banded krait antivenom neutralises 0.6 mg of banded krait (*B. fasciatus*) venom. Australian polyvalent antivenom is raised against black snakes (*Genus pseudechis*), Taipans (Genus: *Oxyuranus*), death adders (Genus: *Acanthophis*), tiger snakes (Genus: *Notechis*) and brown snakes (Genus: *Pseudonaja*). One vial of Australian polyvalent antivenom contains 12,000 units of Taipan antivenom.

The required amount of Indian polyvalent antivenom (i.e., ×1 concentration) for neutralisation of a given amount of *B. ceylonicus* venom was calculated based on the recommended amount of Indian polyvalent antivenom for neutralising a similar amount of *B. caeruleus* venom. For banded krait antivenom, the required amount of antivenom (×1 concentration) was calculated based on the recommendation for neutralising the same amount of *B. fasciatus* venom. In the absence of a species closely related to *Bungarus* in the immunisation mixture of Australian polyvalent antivenom, the required amount of antivenom (×1 concentration) was arbitrarily calculated based on the recommendation for neutralising the same amount of coastal taipan (*Oxyuranus scutellatus*) venom.

### 5.2. Chick Biventer Cervicis Nerve-Muscle Preparation

Chicks (7–10 days old) were humanly killed by CO_2_ inhalation and immediately dissected to obtain two biventer cervicis muscles from each animal. The muscles were then mounted on wire tissue holders at a resting tension of 1 g in 25 mL organ baths maintained at a temperature of 34 °C containing physiological salt solution (composition (mM): 118.4 NaCl, 4.7 KCl, 1.2 MgSO_4_, 1.2 KH_2_PO_4_, 2.5 CaCl_2_, 25 NaHCO_3_ and 11.1 glucose) bubbled with 95% O_2_ and 5% CO_2_. Indirect twitches of the muscle were evoked by stimulating the motor nerve at 0.1 Hz with 0.2 ms pulse duration at a supramaximal voltage (7–15 V). To ensure selective stimulation of the motor nerve, the abolishment of indirect twitches with d-tubocurarine was demonstrated. Tissues were then repeatedly washed with the physiological salt solution to restore the indirect twitches. Contractile responses to exogenous acetylcholine (ACh; HIMedia, Vadhani Industrial Estate, Mumbai, India;1 mM for 30 s), carbachol (CCh; Sigma-Aldrich, St. Louis, MO, USA; 20 µM for 60 s) and KCl (40 mM for 30 s) were then obtained in the absence of nerve stimulation. The preparations were then stimulated for 10 min before adding venom, antivenom or vehicle (i.e., distilled water). For all experiments that examined neutralisation of venom by antivenoms, the antivenom was added to the organ bath 5 min prior to the venom. Organ bath experiments were carried out for a maximum of 3 h, or until twitches were inhibited, and contractile responses of the muscle to exogenous additions of ACh, CCh and KCl were obtained at the end of the experiments.

### 5.3. Experimental Design

Preliminary experiments were conducted to identify concentrations of venom that abolished indirect twitches. Venom concentrations of 0.03, 0.1, 0.3, and 1 µg/mL were selected for further experiments. Based on these experiments, venom concentrations for pre-synaptic neurotoxicity neutralisation and post-synaptic neurotoxicity neutralisation experiments, using antivenoms, were selected. In all neutralisation experiments, antivenom-only experiments were also carried out to ensure that the antivenoms did not affect tissue viability per se.

For pre-synaptic neurotoxicity experiments, ×1 and ×10 concentrations of the antivenoms were used, while ×1 concentrations were used for all post-synaptic neurotoxicity experiments.

### 5.4. Interpretations and Definitions

#### 5.4.1. Neurotoxicity

Full neutralisation, by the test antivenom, of venom-induced neurotoxicity was defined as the absence of a significant difference between the % twitch heights of the venom + antivenom and the control neuromuscular preparations at the end of the observation period. Failure of the test antivenom to neutralise venom-induced neurotoxicity was defined as the absence of difference between the % twitch heights of the venom + antivenom and the venom-alone neuromuscular preparations at the end of the observation period. Partial neutralisation of venom-induced neurotoxicity was identified when the % twitch height of the venom + antivenom preparation was different from both the control and the venom-alone preparations at the end of the observation period.

#### 5.4.2. Pre-Synaptic vs. Post-Synaptic Neurotoxicity

The chick biventer cervicis nerve-muscle preparation is used to distinguish between pre-synaptic neurotoxicity and post-synaptic neurotoxicity by observing the effects of venoms on the contractile responses of the preparation to exogenous ACh and CCh. A significant inhibition of contractile responses to ACh and CCh in the presence of venom compared to the control suggests post-synaptic neurotoxicity, while no significant inhibition of responses is indicative of pre-synaptic neurotoxicity. However, when both pre- and post-synaptic activities are present in a given concentration of a venom, the pre-synaptic activity is often masked by the post-synaptic activity of the venom.

Full neutralisation, by the test antivenom, of post-synaptic venom-induced neurotoxicity was defined as the absence of a significant difference between control and venom + antivenom in contractile responses to both ACh and CCh at the end of the experiment. The absence of neutralisation of venom-induced post-synaptic neurotoxicity was defined as the absence of a significant difference between control and venom + antivenom in both ACh and CCh responses at the end of the experiment. Any other combination of differences between contractile responses to ACh and CCh of venom + antivenom versus venom or control was considered a partial neutralisation of the post-synaptic neurotoxicity.

#### 5.4.3. Data Analysis and Statistics

Indirect twitch responses and responses to exogenous agonists (ACh, CCh and KCl) were measured via an MLT0201 force transducer (ADInstruments Pty Ltd., Bella Vista, NSW, Australia) and recorded on a PowerLab system (ADInstruments Pty Ltd., Bella Vista, NSW, Australia). All twitch and agonist responses were expressed as percentages of their pre-venom +/− antivenom values. An unpaired *t*-test was used to compare the twitch and agonist response of test muscles with the control, as well as to compare the twitch inhibition by two venoms, in venom concentration-dependent neurotoxicity experiments. A one-way ANOVA was used to compare the twitch inhibition and responses to exogenous agonists in all antivenom experiments. All ANOVAs were followed by Tukey’s multiple comparison post-tests. Data are presented as a mean with the standard deviation (SD) or standard error of the mean (SEM) of three to six experiments. All statistical analyses and presentation of data were generated using GraphPad Prism 9 software (GraphPad Software Inc., La Jolla, CA, USA). For all statistical tests, *p* < 0.05 was considered statistically significant. Central tendency was expressed in mean with the Standard Deviation (SD) and Standard Error of Mean (SEM).

## Figures and Tables

**Table 1:** Time taken for 50% decrease in twitch height (t_50_) and 90% decrease in twitch height (t_90_) in chick-biventer nerve muscle preparation by different concentrations of *B. ceylonicus* and *B. caeruleus* venom.

Venom Concentration	Extent of Twitch Inhibition	*B. ceylonicus* (n = 3–6) Mean +/− SD min	*B. caeruleus* (n = 3–6) Mean +/− SD min	Comparison (Unpaired *t*-test, *p*)
0.03 μg/mL	t_50_	115.3 (5.0)	123.0 (8.7)	0.2576
	t_90_	N/A	N/A	
0.1 μg/mL	t_50_	94.0 (8.9)	119.0 (10.0)	**0.0326**
	t_90_	149.3 (1.2)	N/A	
0.3 μg/mL	t_50_	76.3 (6.7)	74.7 (0.6)	0.6881
	t_90_	118.0 (1.7)	150.7 (1.2)	**<0.0001**
0.1 μg/mL	t_50_	58.7 (4.7)	71.0 (8.0)	0.0830
	t_90_	79.7 (4.0)	129.3 (2.1)	**<0.0001**

Notes: N/A: not applicable.

## Data Availability

The original contributions presented in this study are included in the article. Further inquiries can be directed to the corresponding author(s).

## Abbreviations

The following abbreviations are used in this manuscript:

DOAJ	Directory of open access journals
TLA	Three letter acronym
LD	Linear dichroism
NMJ	Neuromuscular junction
ACh	Acetylcholine
CCh	Carbachol
KCl	Potassium Chloride
NaCl	Sodium Chloride
MgSO_4_	Magnesium Sulphate
KH_2_PO_4_	Potassium dihydrogen phosphate
CaCl_2_	Calcium Chloride
NaHCO_3_	Sodium hydrogen carbonate
O_2_	Oxygen
CO_2_	Carbon Dioxide
